# The Development of Myxedema Coma in the Setting of Euthyroid Sick Syndrome: A Diagnostic Dilemma and Review of Management Strategies

**DOI:** 10.7759/cureus.64406

**Published:** 2024-07-12

**Authors:** Shaniah S Holder, Gabrielle Unbehaun, Dejeau P Pyfrom, Abigail Greaves, Atif A Muhammad, Frank Hsu

**Affiliations:** 1 Medical School, American University of Barbados School of Medicine, Saint Michael, BRB; 2 Medical School, Saint George’s University School of Medicine, True Blue, GRD; 3 Medical School, Saint James School of Medicine, Arnos Vale, VCT; 4 Surgery, West Suburban Medical Center, Chicago, USA; 5 Internal Medicine, Insight Medical Center, Chicago, USA

**Keywords:** thyroid pathology, severe sepsis, medical intensive care unit (micu), myxedema coma, euthyroid sick syndrome

## Abstract

Euthyroid sick syndrome (ESS), also referred to as nonthyroidal illness syndrome, is an intriguing condition characterized by dysregulation of thyroid hormones despite normal thyroid gland function. It is diagnosed by low serum triiodothyronine levels, and, in some cases, other thyroid hormones such as thyroxine and thyroid-stimulating hormone may be affected. This condition arises via various physiologic mechanisms and is associated with intensive care unit (ICU) admissions, caloric deprivation, and severe illness. Myxedema coma (MC) is a rare medical emergency with a high mortality rate. It is caused by severe hypothyroidism, resulting in multiorgan failure with features including adrenal insufficiency, thermal dysregulation, and altered mentation. Generally, it is observed in untreated and poorly managed cases of hypothyroidism. However, stress from infections, surgical procedures, and medical comorbidities may precipitate this condition. It is particularly uncommon to see MC arise in the setting of ESS, especially in a patient with no history of thyroid disease, which makes this diagnosis easy to miss. In our case, a 36-year-old female presented with septic shock and was admitted to the ICU, where she subsequently developed ESS and features of MC. This case report aims to explore the risk factors, features, and diagnostic and therapeutic management of these conditions, as well as the diagnostic challenges that arise when these diseases present simultaneously.

## Introduction

The thyroid gland is instrumental in maintaining optimal physiological function by regulating the body’s metabolism. Two of its principal hormones, thyroxine (T4) and triiodothyronine (T3) are primarily controlled by thyroid-stimulating hormone (TSH) secreted by the anterior pituitary gland. Euthyroid sick syndrome (ESS), also known as nonthyroidal illness syndrome, refers to a complex set of medical conditions in patients with illnesses not arising from primary pituitary or thyroidal dysfunction [1,2]. In ESS patients, the most common abnormalities include low T3, increased reverse T3 (rT3), and low TSH and T4 levels. Approximately 10% of hospitalized individuals with ESS exhibit low TSH levels, an abnormality unrelated to thyroid pathology [2]. Several studies have noted that when T4 levels drop below 2-4 µg/dL, the probability of death increases drastically in hospitalized patients [1,2]. Unlike true hypothyroidism, which results in low levels of T3, rT3, and T4 along with high levels of TSH, these distinct patterns help differentiate ESS.

In contrast, myxedema coma (MC) is a rare but severe condition that can arise in the setting of untreated or neglected hypothyroidism, ultimately leading to physiological decompensation [3]. It has a higher predisposition in the female and elderly population, with an estimated mortality rate of 25-60%, even after treatment initiation [4]. This endocrine crisis occurs secondary to infections, trauma, gastrointestinal bleeding, and cerebrovascular accidents. Patients with MC may experience a range of symptoms, including altered mental state, dysthermoregulation, hyponatremia, hypercapnia, and myxedema of the hands and face [5]. While a coma is not a prerequisite for MC diagnosis, it is often present and indicates a poor prognosis [4,5]. Without proper treatment and diagnosis, MC can lead to organ dysfunction and high mortality. Therefore, early symptom recognition and prompt medical attention are essential to minimize the risk of complications.

This case report details the presentation of a young female patient admitted to the intensive care unit (ICU) with septic shock secondary to ventilator-associated pneumonia (VAP), who subsequently developed MC in the setting of ESS.

## Case presentation

A 36-year-old female presented to the emergency department (ED) from her nursing home due to concerns of hypotension and tachycardia. Her past medical history was significant for scimitar syndrome with right lung hypoplasia and subsequent heart failure with reduced ejection fraction (ejection fraction 35-40%), epilepsy, and a malignant neoplasm of the breast with secondary malignant pericardial effusion.

On physical examination, the patient appeared cachectic, and her mental status consisted of eye-opening and ocular movement which was her baseline. Orientation could not be assessed due to the patient being deaf and nonverbal. Heart sounds were appreciated on the right side with no murmurs, gallops, or rubs, and lung auscultation revealed crackles in the left lower lobe. Vital signs were significant for hypotension with a blood pressure of 81/53 mmHg (normal value: ~120/80 mmHg), hypothermia with a temperature of 35.1℃ [95F] (reference range: 36.1°C (97°F)-37.2°C (99°F)), tachycardia with a heart rate of 118 beats per minute (reference range: 60-100 beats per minute), and tachypnea with a respiratory rate of 44 breaths per minute (reference range: 12-18 breaths per minute).

Although her white blood cell count was normal, the procalcitonin level was elevated, measuring 5.92 ng/mL (normal range: <0.05 ng/mL), indicating underlying inflammation and a possible infection. The sepsis protocol was initiated accordingly.

**Figure 1:**
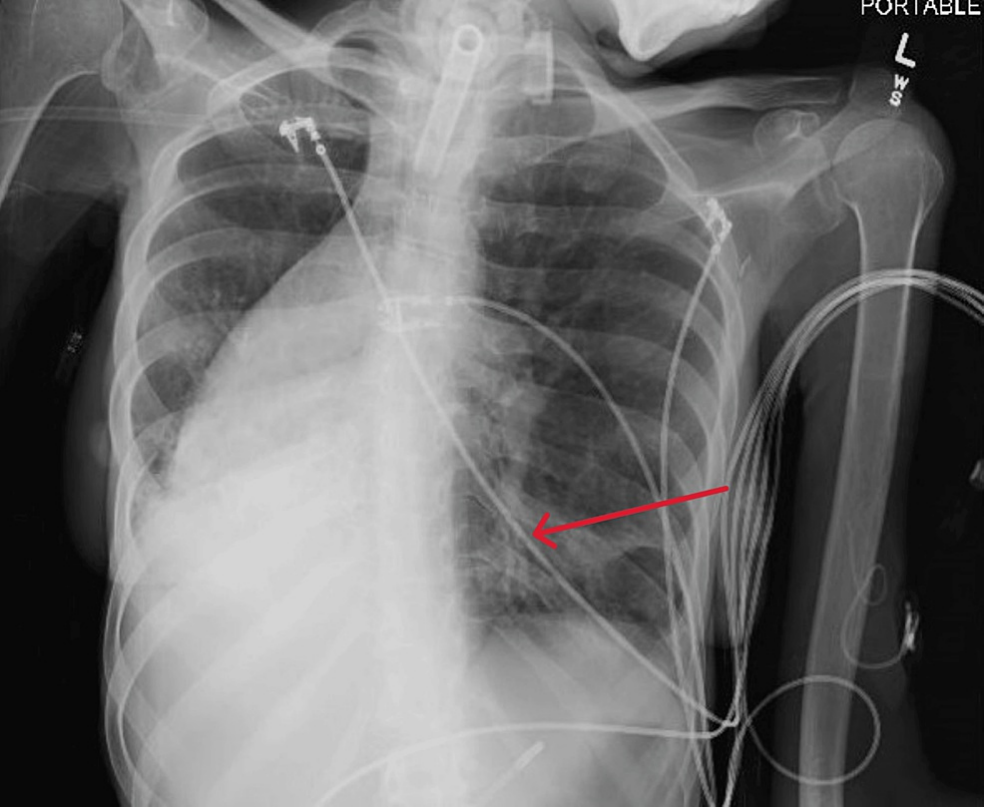
Chest X-ray showcasing a basilar opacity in the left lower lobe (red arrow) and right-sided mediastinal shift.

A chest X-ray revealed extensive lung atelectasis with a mediastinal shift to the right side and a left basilar opacity suggestive of pneumonia. Given her history of a chronic tracheostomy attached to a ventilator, VAP was suspected. Subsequent sputum cultures confirmed the growth of *Proteus mirabilis* and *Acinetobacter baumannii.* Figure 1 highlights the X-ray findings.

Urinalysis revealed pyuria and subsequent cultures showed growth of vancomycin-resistant enterococci. The patient was started on intravenous (IV) fluids and antibiotics and was admitted for further evaluation due to multiple infectious sources.

During the next six days in the general ward, she was managed for chest sepsis. On day six, a rapid response was called due to a seizure episode. The patient’s eyes were rolled back and not tracking finger movements; her oxygen saturation was also decreasing. She was managed with a single dose of IV lorazepam and airway suctioning, along with increasing FiO_2_ on the chronic ventilator, which led to seizure cessation and improvement of oxygen saturation. It was also noted at this time that despite aggressive fluid resuscitation, her hypotension persisted, necessitating the initiation of the vasopressor norepinephrine. The patient was transferred to the ICU.

Despite two weeks of management in the ICU, there was no improvement in the patient’s clinical condition. Her vasopressor requirement increased, necessitating the addition of phenylephrine, vasopressin, as well as midodrine 15 mg every eight hours to reduce vasopressor use. Additionally, she experienced recurrent hypoglycemia refractory to multiple IV injections of 50% dextrose and continuous 5% dextrose drip, and her severe electrolyte imbalance persisted despite continuous replenishment. Table 1 highlights these significant electrolyte imbalances.

**Table 1:** The patient’s abnormal electrolyte values. *: Corrected value; N: normal range

Electrolyte	Patient’s value
Sodium (Na)	119 mEq/L (N = 135–145 mEq/L)
Potassium (K)	2.7 mg/dL (N = 3.5–5.0 mg/dL)
Calcium (Ca)*	7.1 mg/dL (N = 8.5–10.5 mg/dL)
Phosphorus (P)	2.2 mg/dL (N = 2.8–4.5 mg/dL)
Magnesium (Mg)	1.4 mg/dL (N = 1.7–2.2 mg/dL)
Glucose	54 mg/dL (N = 70–100 mg/dL)

Following a comprehensive pharmacological evaluation concerning the low sodium levels, none of the administered medications were identified as etiological agents for the development of the syndrome of inappropriate antidiuretic hormone secretion or hyponatremic states. Furthermore, transient bradycardia was noted, as well as hypothermia with a heart rate in the 50s and an average core temperature of 34.6°C (94.3°F), despite active external rewarming methods. Due to these findings, it was suspected that thyroid dysfunction was the cause of her electrolyte disturbance, vital sign instability, and high vasopressor requirement. Considering numerous risk factors and an MC diagnostic score of 115, the conditions ESS and MC were suspected. Table 2 showcases the patient’s MC score.

**Table 2:** Myxedema diagnostic score criteria in addition to the patient’s score. *: EKG changes including QT prolongation, low voltage complexes, bundle branch blocks, nonspecific ST-T changes, or AV blocks. CNS: central nervous system; EKG: electrocardiogram; SO_2_: oxygen saturation; PO_2_: partial pressure of oxygen; GFR: glomerular filtration rate; MC: myxedema coma

Parameters	Score criteria		Patient’s score
Thermoregulatory Dysfunction	>35°C	0	10
	32–35°C	10	
	<32°C	20	
CNS effects	Absent	0	20
	Somnolent/Lethargic	10	
	Obtunded	15	
	Stupor	20	
	Coma/Seizures	30	
Gastrointestinal findings	Anorexia/Abdominal pain/Constipation	5	15
	Decreased intestinal motility	15	
	Paralytic ileus	20	
Precipitating event	Absent	0	10
	Present	10	
Bradycardia	Absent	0	10
	50–59	10	
	40–49	20	
	<40	30	
Other EKG changes*	10		0
Pericardial/Pleural effusion	10		10 (pericardial effusion)
Pulmonary edema	15		0
Cardiomegaly	15		0
Hypotension (<90/60 mm/Hg)	20		20
Metabolic disturbance	Hyponatremia (<135 mEq/L)	10	20 (Hyponatremia and hypoglycemia)
	Hypoglycemia (<60 mg/dL)	10	
	Hypoxemia (SO_2_ <88, PO_2_ <55)	10	
	Hypercapnia (PCO_2_ >50)	10	
	Decrease in GFR	10	
Patient’s total score	> 60: Highly suggestive/diagnostic of MC		115
	25-59: Suggestive of risk for MC		
	<25: Unlikely to indicate MC		

TSH, T3, T4, and cortisol levels were measured. Labs showed decreased serum concentrations of T3 and T4, with a TSH level within the lower end of the normal reference range. These findings are indicative of late-stage ESS. Furthermore, the comprehensive assessment using the Myxedema Coma Diagnostic Score Tool, which integrates clinical symptoms, vital signs, and laboratory parameters, yielded a score exceeding 60. This score is highly suggestive of, if not diagnostic for MC. In addition to this, the patient’s cortisol level was low indicating a concomitant adrenal insufficiency. Table 3 shows the results of the thyroid function test and cortisol level.

**Table 3:** Cortisol level and thyroid function test results. N: normal value; TSH: thyroid-stimulating hormone; T3: triiodothyronine; T4: thyroxine

Hormone	On admission	Two weeks later
TSH	3.51 mU/mL	0.91 mU/mL (N = 0.4–4.0 mU/L)
Free T3	Not measured	0.6 pg/mL (N = 2.3–4.1 pg/mL)
T4	Not measured	0.6 ng/dL (N = 0.8–1.8 ng/dL)
AM cortisol	15 µg/dL	5 µg/dL (N = 6–23 µg/dL)

She was placed on levothyroxine 200 µg for one day, followed by 50 µg IV daily. Additionally, she received an oral liothyronine 5 mg loading dose, followed by 2.5 mg every eight hours. Hydrocortisone was administered at 100 mg IV every eight hours initially, followed by 50 mg IV every six hours. After one week on this regimen, the patient became more alert, with spontaneous eye opening (which was her baseline). Her vasopressor requirement decreased to only norepinephrine, maintaining an average mean arterial pressure of 68. Furthermore, her bradycardia, hypothermia, hypoglycemic episodes, and electrolyte disturbance resolved. New electrolyte and glucose readings are highlighted in Table 4.

**Table 4:** The patient’s updated electrolyte values. *: corrected value

Electrolyte	Values	Normal values
Sodium (Na)	136 mEq/L	135–145 mEq/L
Potassium (K)	4.3 mg/dL	3.5–5.0 mg/dL
Calcium (Ca)*	8.36 mg/dL	8.5–10.5 mg/dL
Phosphorus (P)	2.2 mg/dL	2.8–4.5 mg/dL
Magnesium (Mg)	3.0 mg/dL	1.7–2.2 mg/dL
Glucose	95 mg/dL	70–100 mg/dL

The plan was to reevaluate the thyroid function and evaluate her adrenal insufficiency. However, given her existing comorbidities and unfavorable prognosis, she coded and expired. Cardiopulmonary resuscitation was deferred in accordance with the patient’s code status.

## Discussion

Thyroid gland activity is controlled by the hormone TSH, which is regulated by the inhibitory effects of thyroid hormones and the stimulatory action of TRH [6]. The thyroid creates T4 and T3 from ingested iodine, which is oxidized and incorporated into thyroglobulin (Tg) to form monoiodotyrosine (MIT) and diiodotyrosine (DIT) [7]. MIT and DIT combine to form T3, and two DITs combine to form T4 [7]. Tg undergoes proteolysis, and these lipophilic hormones are secreted into circulation bound to transport proteins and carried to target tissues [6]. T3 and T4 bind to thyroid receptors in tissues, which act as transcription factors, leading to cell-specific responses and gene activation [6]. These receptors have a higher affinity for T3, so deiodinases convert T4 to active T3 or inactive rT3 [7].

The reduced peripheral conversion of T4 to T3 is the main pathophysiologic mechanism behind the development of ESS [7]. Another proposed mechanism involves the production of cytokines such as interleukin-1, interleukin-6, and tumor necrosis factor-alpha, which decrease the activity of deiodinases and the binding capacity of T3 receptors. Additionally, these cytokines may affect the hypothalamus and pituitary gland, inhibiting the production of TSH, TRH, Tg, T3, and thyroid-binding globulins [8]. Hospitalized patients, especially those who are severely ill, have a higher incidence of abnormal thyroid hormone levels unrelated to thyroid pathology. This phenomenon is commonly observed in conditions such as acute myocardial infarction, heart failure, pneumonia, sepsis, and malignancies [7]. Early-stage ESS is characterized by low plasma levels of T3, while late-stage ESS is distinguished by low T3, T4, and TSH mimicking secondary hypothyroidism [2,8]. Due to similar features, coexisting ESS may mask an underlying diagnosis of hypothyroidism which can subsequently lead to MC.

MC is an exceedingly rare, life-threatening progression of severe hypothyroidism, causing profound disruptions and slowing of the body’s physiological processes. Occurring in only 0.22 cases per million per year in the Western world, this endocrinologic emergency has a mortality rate of 25-60% due to the widespread effects of thyroid hormone on the body [9,10]. While the diagnosis of MC is made clinically, the key features of the disease include altered mental status and hypothermia caused by a precipitating event in the setting of hypothyroidism [11]. Other symptoms can include hypotension, decreased gastrointestinal motility, hyperventilation, severe electrolyte abnormalities, and nonpitting edema [9,11]. Although it is most commonly seen in patients with chronic autoimmune thyroiditis, MC can be triggered by any form of hypothyroidism exacerbated by a stressor such as an infection, burns, trauma, or medications such as amiodarone or lithium [9,10,12].

The diagnosis of ESS is established when there are low serum total T3 levels and increased rT3 levels. Both decreased T3 and T4 are seen in critically ill patients, and low T4 is associated with a poor prognosis [8]. MC can be fatal; therefore, prompt diagnosis and treatment are essential to reduce mortality. An accurate diagnosis involves a thorough history, physical examination, and laboratory evaluation. Lab results may reveal anemia, hypocortisolemia, hyponatremia, and hypoglycemia [11]. Thyroid function test findings include low serum free T4 and high serum TSH; however, TSH may be low or even normal due to the presence of central hypothyroidism and concomitant ESS, respectively [11].

MC in the setting of ESS can prove to be a diagnostic challenge, as the hallmark elevated serum TSH of severe hypothyroidism is camouflaged [9,11]. ESS results in central hypothyroidism, leading to normal or low serum TSH, which subsequently results in secondary thyroid failure with low serum total and free T4 and especially low (<25 ng/mL) serum T3 levels [8]. Additionally, patients with ESS may not exhibit the physical findings classically associated with hypothyroidism, further complicating the diagnostic picture. In this case, the patient’s free T3 and T4 were decreased, and TSH was borderline normal, signifying a transition into late-stage ESS. Due to the severity of her clinical deterioration despite the resolution of her sepsis, as well as the thyroid and cortisol hormone abnormalities, this confirmed a mixed picture of ESS and MC.

Treatment for ESS involves managing the underlying medical illness, and thyroid hormone replacement is generally not necessary [8]. However, in this case, the patient had MC in the setting of ESS requiring hormone replacement therapy. Patients with MC should be admitted to the ICU for close monitoring of vital signs and electrolytes until the underlying cause precipitating MC has been identified and addressed [9]. As infection is one of the most common causes precipitating MC, a complete infectious workup should be conducted, including urine and blood cultures, along with a chest X-ray [9,12]. If a specific infection source cannot be identified but there is a high level of suspicion for an infectious cause, physicians may consider broad-spectrum antibiotics. During the interim, the patient should be treated presumptively with IV thyroid hormone and stress-dose steroids [12]. Special consideration for the patient’s cardiovascular status should be taken during this time due to concern for myocardial infarction or arrhythmias that may be triggered by thyroid hormone infusion [9,12].

While rare, MC presents a significant mortality risk to those affected. In severely ill patients, ESS can trigger MC and can obfuscate the diagnosis by masking the salient TSH elevation. Physicians should have a high degree of suspicion in patients presenting with mental status changes in the setting of critical illness, as early intervention with intensive care can significantly impact the survival rate of these patients.

## Conclusions

ESS is observed in many critical care patients and is characterized by a thyroid hormone imbalance despite normal gland physiological function. Distinguishing findings include low T3 levels in the early stage, with subsequent decreases in T4 and TSH levels in the late, more severe stage of this disorder. Although ESS does not directly cause MC, underlying stressors such as infection, malignancy, and shock can lead to allostatic overload, contributing to the physiological decompensation characteristic of MC.

In the setting of ESS, the normally elevated TSH that is characteristic of MC may be masked, leading to a delay in diagnosis and treatment. This delay is associated with swift clinical deterioration and a high mortality risk. To mitigate this risk, critical care physicians must maintain a high index of suspicion for MC, allowing for timely diagnosis. Confirmatory diagnostic tests include total T3 (with or without rT3), free T4, TSH, and cortisol levels. Prompt treatment with thyroid hormone replacement therapy and cortisol supplementation can improve prognosis in critical care patients and lead to significant improvement in organ function.
